# Injury patterns of suicide attempts in the head and neck area—a retrospective analysis over 15 years

**DOI:** 10.1007/s00405-024-09138-2

**Published:** 2024-12-19

**Authors:** R. Lehner, R. Lochbaum, T. K. Hoffmann, J. Hahn

**Affiliations:** Department of Otorhinolaryngology, Head and Neck Surgery, University Hospital of Ulm, Frauensteige 12, 89075 Ulm, Germany

**Keywords:** Suicide attempts, Head and neck, Strangulation, Gunshot wound, Gender medicine

## Abstract

**Purpose:**

Suicide attempts may involve various parts of the body with different severity grades and therefore represent a multidisciplinary challenge. The head and neck region is highly vulnerable to severe self-inflicted injuries, yet literature on this topic remains limited.

**Methods:**

A retrospective analysis was performed of patients with suicide attempts in an Otorhinolaryngology (ORL) department of a tertiary referral hospital over a 15-year period. The aim of the study was to analyse their clinical course and injury patterns.

**Results:**

70 patients were included (m: 42/70; f: 28/70). The mean age at suicide attempt was 43.7 years. Women were significantly younger than men (p = 0.046). Seven injury types were differentiated: strangulation (44.3%), stabbing (17.1%), jumping from a height and firearm use (10.0% each), jumping in front of a moving vehicle and ingestion of acids/bases (7.1% each) as well as ingestion of pills (4.3%). Men were dominantly involved in strangulation (14/42; 33.3%) and stabbing (11/42; 26.2%), whereas women appeared with strangulation (17/28; 60.7%) and tablet ingestion (3/28; 10.7%). Men required ORL-specific surgical care significantly more often than women (43.9% vs. 7.1%; p < 0.001). Men chose “violent” methods more frequently than women (90.5% vs. 46.4%; p < 0.001). Women were more likely to receive psychiatric treatment (p = 0.0011).

**Conclusions:**

Violent suicide attempts were more common in males and therefore required more often surgical intervention. Soft attempts and psychiatric diagnoses were more often associated with female gender. Routine laryngoscopy is recommended within 24h after the initial trauma. All individuals were successfully treated in an interdisciplinary setting and survived with moderate morbidity.

## Introduction

Suicide and suicide attempts pose significant challenges for medical experts. Not only is the diagnosis and treatment of the underlying cause a difficult undertaking, but the immediate consequences can lead to severe impairments and injuries. Addressing these issues may necessitate the involvement of a highly specialized and interdisciplinary team.

According to governmental sources, 10.300 people died in 2023 due to suicide in Germany [1]. This being a decrease of 3.1% in the last decade, compared with the numbers from 1980 it’s an even bigger decline, by 44.2% [2]. The gender distribution remained constant (73% male vs. 27% female). Still, suicide accounts for 1% of the total deaths in Germany. Worldwide, more than 720,000 people commit suicide each year (World Health Organization, WHO [3]). This high number has made it a hot topic for the WHO since the 1950’s [4]. Unfortunately, the WHO does not receive data about suicide attempts. Experts assume that the number of suicide attempts is significantly higher, some data suggests a 10–30 times greater risk [5–9]. Suicide is a global phenomenon that occurs in every country and affects men as well as women. It occurs at every age and is the third leading cause of death in young adults [3]. The majority of cases is related to psychiatric disorders [9]. After the first WHO world suicide report in 2014, the WHO has launched multiple projects to raise awareness and underline the seriousness of this matter [10].

Due to the wide range of injury patterns, almost every medical discipline can be involved in the diagnosis and treatment of patients. Analysing the characteristics of suicide attempts can provide essential information, such as the risk of further attempts. A US study found that a younger age at the first suicide attempt and a lack of psychological care after the initial attempt were associated with subsequent attempts [11]. An individual’s exposure to a suicide attempt by any means increases the risk of attempting one [12].

The role of Otorhinolaryngology (ORL) plays a major role in the management of self-inflicted injuries, especially considering that the head and neck region, with its vital structures, presents a specific "target point". 30% of all suicide attempts target the head and neck region [13]. Common methods include strangulation, jumps from a height and self-inflicted stab or gunshot wounds, though the chosen method depends on the specific world region [13–15]. Most authors do not distinguish between the localization of the self-inflicted injuries, still, all penetrating neck wounds are considered dangerous with the instant need of proper evaluation and often intervention [16]. International guidelines on the evaluation and care of suicidal neck wounds are not established, but there are general guidelines to handle traumatic injuries, though without especially considering suicide attempts [17]. The surgical wound classification can be helpful as well [18].

Some authors distinguish between “soft” and “violent” attempts, although there is no universally accepted definition. In general, gender specific differences concerning the age, method and number of suicide attempts exist. Women present higher rates of attempted, men higher rates of completed suicides, which is a factor that seems to remain constant over lifetime [9, 19, 20]. Furthermore, the male gender is commonly associated with “violent” suicide attempts, but clinical and temperamental characteristics must also be taken into account [21]. This *gender paradox* has been known for decades [22, 23]. Cultural expectations about gender itself across the globe may be responsible for this phenomenon [24, 25]. Especially the gender specific approaches in handling psychological distress in relation to the socially established gender norms must be considered [26].

After a strangulation attempt, depending on the individual case, harm to the cervical vessels and/or the larynx can occur. The resulting consequential damage can range from superficial skin lesions to laryngeal structural injuries compromising speech and swallowing, and even major swelling—sometimes secondary or caused by venous bleeding—leading to acute or delayed dyspnea. The latter can be life-threatening. An appropriate and instant diagnostic is inevitable. Computed tomography (CT) is the “workhorse of traumatic airway” for it being able to identify foreign bodies as well as big and small injuries [27]. The clinical examination, if possible, specifically a laryngoscopy performed by a specialist, is necessary to determine the current status and estimate the further course. This underscores the importance of otorhinolaryngologists in the diagnosis of suicide attempts by strangulation. The data from other studies is very rare [28–30]. Awareness of correlations between external cervical injuries of the skin and/or symptoms like hoarseness with laryngeal injuries requiring special monitoring—such as edema or hematoma—could facilitate assessments by physicians who are unable to perform laryngoscopy. Vocal cord dysfunction and dysphagia can be also relevant in the diagnostic of suicidal patients [31]. Not only the ability to conduct a proper examination, but even further the surgical skill in the head and neck area make otolaryngologists a vital part. Airway security and reconstruction along with facial reconstruction and emergency bleeding management lies within the specialty of otolaryngologists [32–34]. Although, multiple guidelines exist concerning suicide prevention, in Germany a S3-guideline is currently in preparation, none are to be found in the management on self-inflicted head and neck injuries [35, 36].

The present study was conducted with the aim of gathering initial structured data on suicide attempts with implications for the ORL field. The main focus was on collecting patterns of injuries along with corresponding risk factors. As such, it was hypothesized that the gender paradox could also be demonstrated in this cohort. As a result, men would have more severe injuries and more frequent need for interventions.

## Material and methods

This study was conducted as a retrospective observational cross-sectional study.

The investigation and analysis were carried out at the ORL Department of Ulm University Medical Center between 01/2007 and 10/2022 and thus spans almost 15 years. All relevant data was obtained in this timeframe. All patients who had been treated after a suicide attempt were included in the analysis. There was no age or gender restrictions, also both in- and outpatients were included, with no distinction made between those primarily treated at the ORL department and those seen on a consultative basis. Ever attempt was included, there was no limitation considering the damage mechanism. The clinical examination and diagnosis were consistently conducted by an otorhinolaryngologist from the department. Patients who had undergone a suicide attempt before the data collection and were at that time not examined by an otorhinolaryngologist were excluded.

Obtained patients’ characteristics included the age (meaning the age at the attempt) and gender distribution as well as the knowledge of previous or current mental illnesses and the month in which the attempt was undergone.

Furthermore, the individual methods of the attempts were obtained and categorized. Two categories were created to classify “violent” and “soft” suicide attempts. “Violent” attempts included methods that would normally lead to severe injuries, for example to penetrating neck or face injuries [16]. Therefore, stab wounds, use of firearms, jumping from a great height and in front of a moving vehicle were classified as “violent”. “Soft” attempts were more likely to remain the external appearance largely intact [37]. Ingestion of pills and other poisonous substances were put in this category. This classification generally aligns with other publications on suicides, but as mentioned earlier, there are minor differences [38]. Strangulation could be represented in both groups depending on the used material based on the same argumentation as mentioned above (e.g. a wire or cable was accounted into the “violent” category, a scarf into the “soft” one; see Table 1).

**Table 1:** Differentiation of “violent” and “soft” methods

“Violent” methods	“Soft” methods
Stabbing	Pills
Firearm use	Ingestions of acids/bases
Jumping from a Height	
Jumping in front of a moving vehicle	
Strangulation	
E.g. rope, wire, belt…	E.g. scarf, other clothes, bandages…

Moreover, the injury patterns, ORL-specific diagnostics and treatments were analysed. The first including the different symptoms with which the patients presented themselves, including both subjective and objective symptoms. Diagnostics concentrated on objective pathologies obtained by the examining doctor, such as laryngeal pathologies. Injuries were classified into 4 grades. Visible neck injuries were differentiated in hematoma (I°), superficial cuts (II°), deep cuts (III°) and mutilating-like injuries (IV°). Laryngeal (and tracheal) pathologies were categorised in: swelling (I°), hematoma (II°), fracture (III°) and total separation (IV°) Treatment consisted of surveillance and/or necessary surgery. As imaging was not performed in every case, the analysis of radiological findings was not part of the study.

The retrospective analysis was conducted using the department’s electronic medical records, which has been the standard of documentary since 2006 thus explaining the timeframe between 2007 and 2022. This one-of-a-kind tool (which is not commercially available) is mainly used in the ORL-Department and contains all relevant data documented in the department, such as anamneses, clinical examinations, surgery notes. It is linked with the general software of Ulm Medical Center (SAP) and therefore discharge papers and summaries from other departments could be evaluated as well. One exception must be mentioned. There is a limitation to the psychiatric reports which can only viewed by the psychiatric department.

Data was collected and statistically analysed using Microsoft Excel (version 1808), Prism 5 and SPSS (version 28.0.1.0). Descriptive and nonparametric statistical analyses were performed. For group comparison Fisher’s exact test was mainly used, for mean analysis the Kruskal–Wallis-Test was applied, if possible. A p-value of 0.05 or less was considered statistically significant.

Possible drawbacks of this study are the retrospective character and the categorization of the different suicidal methods in “violent” and “soft”. Retrospective analyses can present great differences in the data collection, so incomplete data in individual cases can be possible. To ensure a certain standard, all available information, including photographs and documents from other departments, was carefully analysed. As mentioned above, there are no generally approved and consistent classification of the different suicide methods. As long as this remains this way all studies on this topic present a minor weakness.

Ethics approval of the local ethical committee was obtained beforehand without the need of written consent due to the retrospective approach (application number: 61/23).

## Results

### Clinical and demographic features

**Fig. 1:**
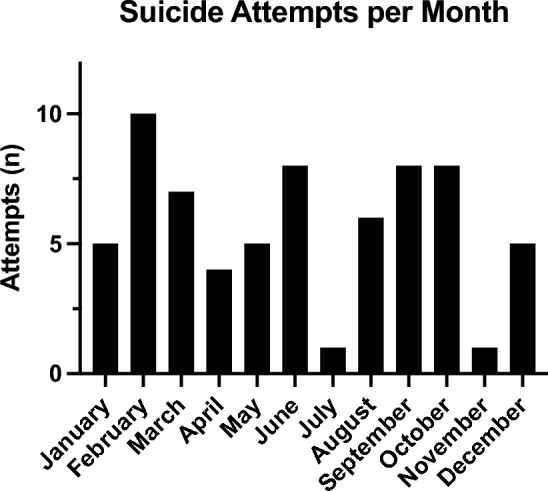
Total numbers of suicide attempts in each month

In total, 70 patients were included (m: 42/70 [60%]; f: 28/70 [40%]). The mean age at the suicide attempt was 43.7 years (m: 47.8 y [11–87 y]; f = 37.6 y [14–93 y]). Psychiatric diagnoses were known in 14 males (14/42 [33.3%] and in 16 females (16/28 [57.1%] of the cases, a total of 30/70 [42.9%]. Most suicide attempts were undergone in February. July and November had the least attempts (Fig. 1).

27/70 [38.6%] of patients presented without any specific symptoms or relevant clinical injuries. 15/70 [21.4%] complained about pain, 6/70 [8.6%] presented with hoarseness, 3/70 [4.3%] with stridor. 14/70 [20.0%] had fractures of the facial bones. 13/70 [18.6%] showed hematoma and/or tissue damage. 10/70 [14.3%] displayed laryngeal and/or tracheal trauma. There was one case of a complete tracheal rupture.

### Injury mechanisms and severity

Seven different methods of suicidal attempts were distinguished: (1) strangulation (31/70 [44.3%]), (2) stab wounds (12/70 [17.1%]), (3) jumping from a height and (4) use of firearms (7/70 [10.0%] each), (5) ingestion of acids/bases and (6) jumping in front of a moving vehicle (5/70 [7.1%] each) and (7) pills (3/70 [4.3%]).

**Fig. 2:**
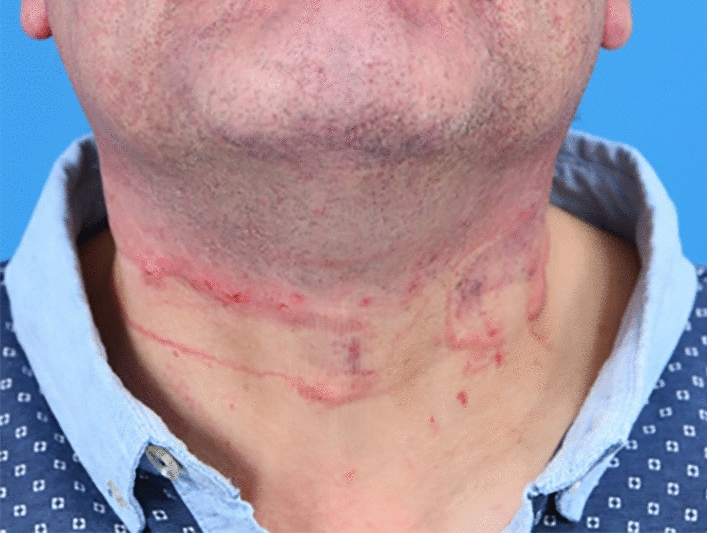
Patient with visible strangulation marks after a suicide attempt using a rope

Visible neck injuries were apparent in 29/70 [41.4%] (see in Fig. 2 an example after a strangulation attempt) of the cases, laryngeal pathologies were diagnosed in 10/70 [14.3%] of patients. The majority of neck injuries were grade I° and II° 21/29 [72.4%]. Grade III° and IV° therefore accounted for 8/29 [20%]. 3/10 [30%] of laryngeal pathologies were graded as I° and II°, 7/10 [70%] as III° and IV°.

Two categories were created to classify “violent” and “soft” suicide attempts, as mentioned before. In total, “violent” attempts were more common (51/70 [72.9%]) and led to more severe injuries, although with no statistical significance (p = 0.1736; 23 cases had to be excluded due to unclear data). The severity of the visible injury on the outside did not correlate with endolaryngeal pathologies (p = 0.3195; 21 cases had to be censored due to missing data).

### Methods, inhouse treatment and the need for surgical interventions

Surgery was necessary in 21/70 [30.0%] of the cases, 2 of those underwent the surgery in an interdisciplinary setting. 12/70 [17.1%] patients required instant endotracheal intubation and therefore therapy and surveillance in an ICU. 6/10 [60.0%] of the patients with laryngeal/tracheal trauma required surgery.

19/70 [27.1%] of the patients were mainly treated by the ORL department, only 2/70 [2.9%] did present themselves directly. 50/70 [71.4%] were seen on a consultative basis, women more often than men (25/28 [89.3%] vs. 25/42 [59.5%]; p = 0.0076). Only one patient was seen for audio diagnostics. 38/70 [54.3%] did have the first contact in other departments (30/42 [71.4%] men vs. 8/28 [28.6%] women; p = 0.0011). 30/70 [42.9%] were sent from the psychiatric ward (11/42 [26.2%] men vs. 19/28 [67.9%] women; p < 0.001). The following addresses the specific injury patterns and precise tasks of the otorhinolaryngologists regarding diagnostic and treatment in more detail:

1. Strangulation (31/70 [44.3%]): 6/31 [19.4%] were mainly treated by the ORL department, 25/31 [80.6%] were seen on a consultative basis. 22/31 [71.0%] were presented by the psychiatric ward, 8/31 [25.8%] by other departments or peripheral hospitals. 1/31 [3.2%] of the patients had their first medical contact at the ORL department. When differentiating between violent and soft methods, 6/20 [30.0%] of patients after a violent strangulation attempt had to be treated directly by the ORL department whereas 8/8 [100%] of soft attempts were seen as on a consultative basis.
2. Stab wounds (12/70 [17.1%]): 7/12 [58.3%] of patients suffering from stab wounds had to be treated primarily by the ORL department, all of which had to undergo surgery (7/7 [100%]). 5/12 [41.7%] of patients were seen on a consultative basis. 4/12 [33.3%] were sent by the psychiatric department, 7/12 [58.3%] by others. 1/12 [8.3%] was presented directly at the ORL department.
3. Jumping from a height (7/70 [10.0%]): 1/7 [14.3%] of patients were primarily treated at the ORL department, 6/7 [85.7] were seen on a consultative basis. 7/7 [100%] were sent by other departments.
4. Use of firearms (7/70 [10.0%]): After use of firearms 7/7 [100%] of the first medical contacts took place in other departments or hospitals. 4/7 [57.1%] were transferred to the ORL for primarily treatment, all of which had to undergo surgery (4/4 [100%]). 3/7 [42.9%] of patients were seen on a consultative basis.
5. Ingestion of acids/bases (5/70 [7.1%]): 5/5 [100%] of examinations took place on a consultative basis. 1/5 [20.0%] of patients were sent by the psychiatric ward, 4/5 [80.0%] by others.
6. Jumping in front of a moving vehicle (5/70 [7.1%]): 4/5 [80.0%] of patients were seen on a consultative basis, 1/5 [20.0%] were treated mainly at the ORL department. 1/5 [20.0%] of patients were sent by the psychiatric ward, 4/5 [80.0%] by others.
7. Pills (3/70 [4.3%]): 3/3 [100%] of patients were seen on a consultative basis, one of them only needed a hearing test. 2/3 [66.7%] were presented by the psychiatric department, 1/3 [33.3%] by others.

Strangulation attempts represented the largest subgroup. In this case, women were significantly more likely to be presented by the psychiatry (p = 0.003). In comparison with the other groups in total, men were presented more often by other departments (p < 0.001).

### COVID-19

56/70 [80.0%] of suicide attempts were undertaken before 2020, spanning over 12 years since the first documented case in this study. 14/70 [20%] of the attempts took place between 01/2020 and 10/2022. This resulted in an average of 4.67 attempts per year before Covid and after Covid. Due to the small group size a sufficient Kruskal–Wallis-Test was not possible to apply.

### Comparisons of genders

**Fig. 3:**
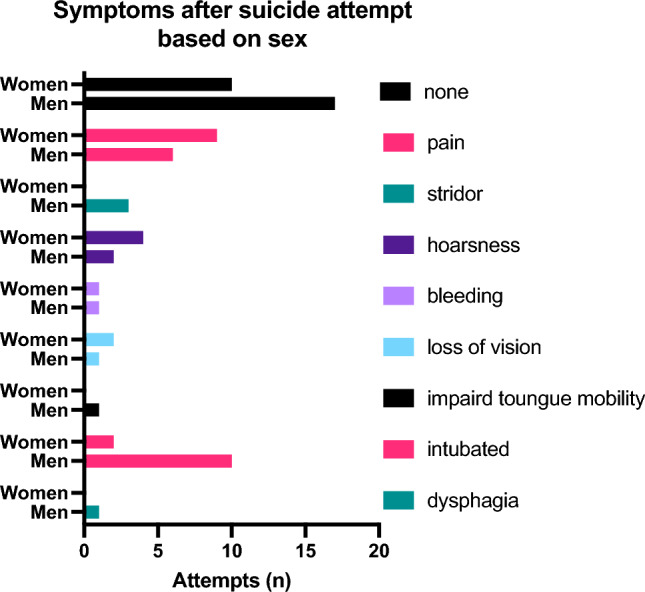
Total numbers of suicide attempts with initial symptoms at first ORL-contact broken down by sex

**Fig. 4:**
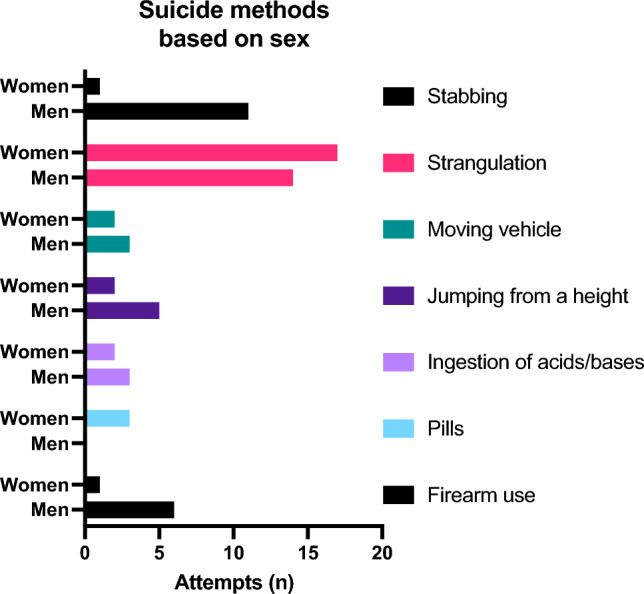
Total numbers of suicide attempts by methods and sex

Women undertook the suicide attempt significantly younger than men (p = 0.046, median: m: 51 y, f: 35 y). In Fig. 3 the gender specific symptoms are shown. When comparing the different methods, the most common ones in women were strangulation (17/28 [60.7%]) and taking pills (3/28 [10.7%]); in men it was strangulation (14/42 [33.3%]), followed by stab wounds (11/24 [26.2%]). Men chose “violent” methods more frequently than women (38/42 [90.5%] vs. 13/28 [46.4%]; p < 0.001). 3 cases of strangulation had to be excluded due to incomplete data (Fig. 4).

Men had to undergo ORL-specific surgery significantly more frequent than women (19/42 [45.2%] vs. 2/28 [7.1%]; p < 0.001). Of the 50 consultations, as mentioned above, women represented the majority (25/28 [89.3%] vs. 25/42 [59.5%]; p = 0.0023). Men on the other hand were more likely to be treated in other departments first (30/42 [71.4%] vs. 8/28 [28.6%]) and women were sent from the psychiatric ward significantly more often (19/28 [67.9%] vs. 11/42 [26.2%]); p < 0.001). Furthermore, men required endotracheal intubation and ICU-treatment more often than women but with no statistical significance (10/42 [24.4%] vs. 2/28 [7.1%]; p = 0.1059).

## Discussion

### Interpretation and earlier research

**Fig. 5:**
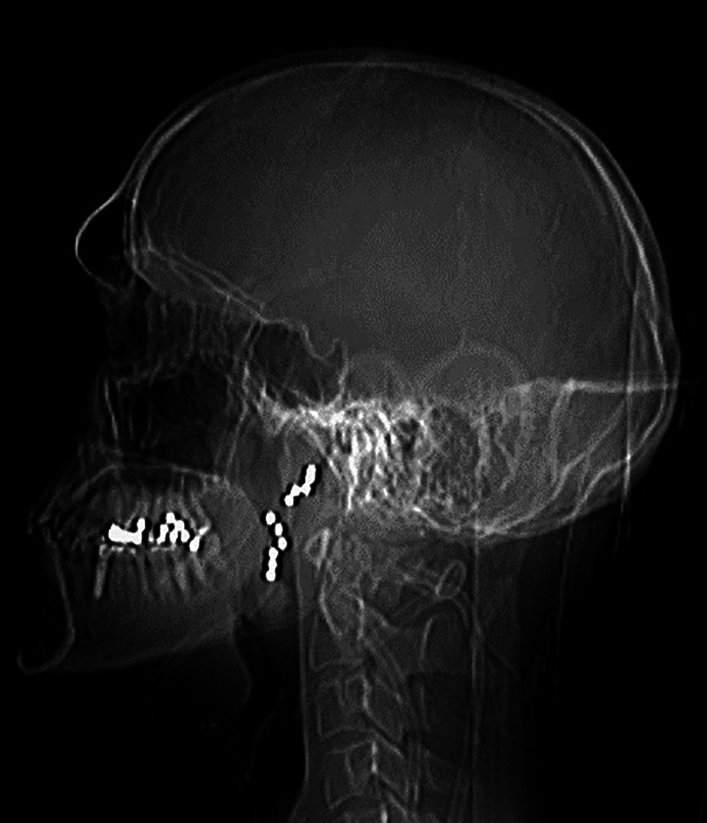
X-ray head after suicide attempt with a shotgun showing multiple shotgun pellets

**Fig. 6:**
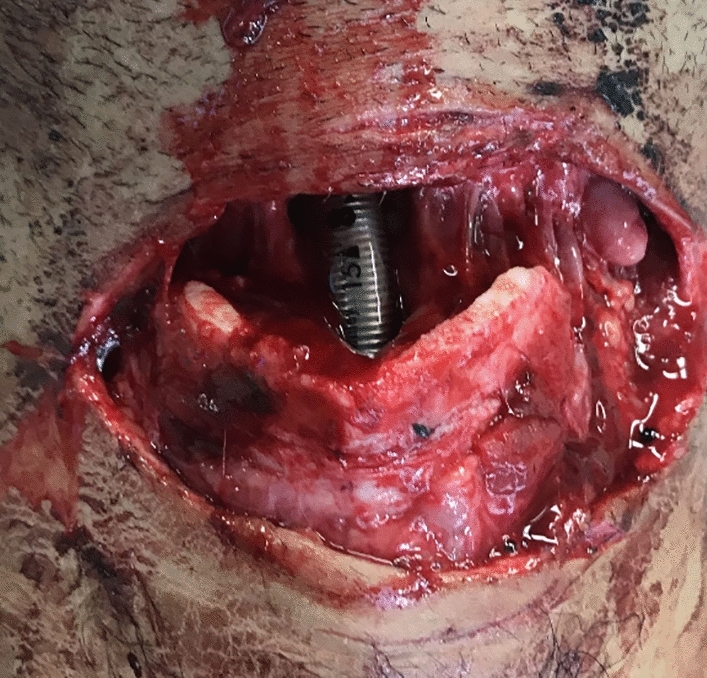
Patient after self-stabbing with a knife showing an anteriorly opened trachea and the inserted tracheal tube

The presented collective included men and women of any age group. This is consistent with current literature, describing suicide a worldwide phenomenon affecting any age, though with differences between countries, regions, gender and socioeconomic factors [9]. With 42.9% of known psychiatric disorders our group is underdiagnosed in this regard. A correlation between suicides and disorders is shown in 87.3–98% of the cases [39]. This circumstance might be mainly explained since only suicide attempts were accounted for and due to the retrospective character of the study which can produce incomplete information. Nevertheless, woman represent the majority in this group with 57.1%. Mental health and its influencing factors vary vastly between men and women [40]. Depression for example is an important comorbidity in both genders but is very much underdiagnosed in men due to a lack of self-reporting such symptoms [41, 42]. The traditional role of men and masculinity resembles a major risk factor for male vulnerability [43]. Men are expected to represent strength, embody resilience, suppress emotional vulnerabilities, and prioritize duty over personal desires, which can cause enormous individual stress [44]. Women were more likely to be treated at the psychiatric ward (p = 0.0011), which emphasizes this difference. To further describe the gender differences, there is a worldwide known peculiarity known as the *gender paradox*, which states that women are more likely to attempt suicide, but men are more likely to complete it [24, 45]. Suicide attempts by men are generally associated with violent methods and thus requiring an intense therapy [46, 47]. The differentiation between violent and soft methods is very common in literature. This can be supported by our results, as men were significantly more likely to use violent methods (p < 0.001) and more likely to require ORL surgical treatment (p < 0.001). Examples for violent methods can be found in Figs. 5 and 6. In the literature, even uncommon weapons such as crossbows or spears were used by men [48, 49]. Concerns about the bodily appearance after death might explain women’s trend to less violent methods, whereas manhood is generally associated with aggression [50].

Cultural differences also impact suicidal behavior differently. Especially individualism and collectivism take part in the construction of social norms. While individualism emphasizes autonomy and self-expression, collectivism focuses on group cohesion and shared responsibilities. These values create different reactions to social issues, including mental health [51].

By definition, it is no surprise that violent attempts produced most of the severe injuries (72.9%), even though a statistical significance was not reached. The seriousness of a nonfatal suicide attempt and the following necessity for medical interventions must be taken seriously because it can be related with more severe psychopathologies and the high risk of another attempt in the future [52, 53].

To enable comparisons with future research, specific criteria for assessing the severity of suicide attempts could be established. For example, distinguishing between ‘soft’ and ‘violent’ attempts could be done by using criteria such as the presence of bruising or penetration wounds as cut-off points. These criteria were not applied in this retrospective study due to variability in the timing of patient examinations. While most patients presented on the day of the attempt or the following day, there were cases where psychological or general stabilization was required first, delaying the ORL examination. This delay may have affected the visibility of injuries. However, such criteria could be incorporated into future prospective studies.

With injuries to the head and neck region secondary airway obstruction is a possible risk even in blunt traumas. Those types of injury are rare but may be life-threatening, especially when penetrating [54, 55]. Penetrating wounds require, as the name suggests, always an intervention. Blunt traumas on the other hand pose a different problematic circumstance, as it is difficult to predict complications. One approach to the evaluation of internal pathologies, particularly of the larynx, is to draw conclusions from the external severity of the injury to the internal findings. Unfortunately, the severity of the visible injury on the outside did not correlate with endolaryngeal pathologies (p = 0.3195). Thus, underlining the importance of laryngo-/pharyngoscopy. Interestingly, in many publications about suicide attempts like strangulation and near-hanging, ORL examination is not even mentioned [56]. Most of the patients (71.4%) were seen on a consultative basis which can be interpreted as a relevant concern of laryngeal/pharyngeal complications. At first, the fact that only 2.9% of the patients had been directly treated by the ORL department may come as a surprise. However, this is due to the local infrastructure, as several locations are established at Ulm University Medical Center and serious and possible life-threating injuries are treated first in an interdisciplinary shock room. The shock room was therefore accounted as the first place of treatment. The fact that more men were initially treated in other departments (mainly in the shock room; p < 0.001) can be used to further emphasize the relationship between men and violence. For most of those who had attempted strangulation, it can be assumed that they were receiving psychiatric treatment (71.0%; 31.4% of all patients), with the majority being women (72.7%; p = 0.003). This highlights the critical need for caution and comprehensive assessment in psychiatric treatment settings, even though strangulation only accounts for a small fraction of self-harm in psychiatric inpatients [57, 58]. Given the vulnerability of these individuals, especially those with severe psychiatric conditions, careful monitoring, early intervention, and tailored care plans are essential to address the underlying mental health issues and reduce the risk of further harm [59].

A seasonal variation, as described in literature with a peak in the spring, could not be detected in this study [60]. There is no explanation for this and thus was classified as an unusual finding. The limited data where the main focus was set on the ORL department might be responsible for this event. Further investigations with a broader approach could help explain this in the future.

The COVID-19 pandemic did lead to higher numbers of depression [61]. As a result, higher rates of suicides and suicide attempts are plausible. In current literature there are studies underlining this statement [62]. However, reviews suggesting no impact haven been published as well [63]. Our study seems not to have had any immediate effect, though a statistical analysis was not properly done due to the small group size. These finding have been reproduced in German studies, some even with a decrease in attempts, though specific regional could not be ruled out [64, 65]. Furthermore, vulnerable groups, such as adolescences, are associated with higher attempt rates [66, 67]. A further analysis with regard to age was not expedient, as our collective was only one part of the total number of cases at the medical center. Nevertheless, further interdisciplinary studies in the future may alter the current statements.

### Laryngoscopy

As mentioned before, laryngeal trauma can lead to severe complications. ORL-specialists’ expertise makes them particularly skilled for performing laryngoscopies. Indirect or flexible endoscopy is easily done with minimal risks. The extent of trauma and the patient’s current condition determine the further procedure. The initial evaluation follows the outlines of the *Advance Trauma Life Support* guidelines [68]. The main priority is the establishment of a secure airway, if that is given further diagnostics are possible [69]. Only 10% of our patients presented laryngeal pathologies but 60% of those had to undergo surgery. In our opinion, this justifies a general recommendation for laryngoscopy within 24h after the initial trauma, even in seemingly *unspectacular* cases.

### Limitations

Retrospective studies always present the risk of missing information which was also the case in our analysis. Unfortunately, some data was missing concerning one of the main aspects, the visible neck injuries as well as the laryngeal pathologies. In those cases, the documented data was inconclusive. Therefore, this statistical analysis must be looked at cautiously. Three cases of strangulation had to be censored and could not be categorized in violent or soft methods, the reason being that the used material was not documented. The definition of said methods are mentioned above. The definition itself may lead to a bias when considering the penetrating neck injuries. A more comprehensive evaluation was limited by several factors inherent in the nature of this study. The focus on an ORL perspective meant that not all cases from the medical center could be included. Particularly psychiatric evaluations and fatal suicide attempts were outside the scope of the study. In addition, the catchment area could not be clearly defined, which further limited the ability to make a national comparison or analysis of cases.

## Conclusion

Head and neck trauma of any kind always requires a proper physical examination and surveillance because severe complications are possible. The necessity for instant airway protection in 17.4% makes these injuries an interdisciplinary challenge. There is no doubt about the advantages of emergency imaging to evaluate bone fractures or arterial dissection [70]. The risk of secondary airway obstruction can be assessed based on laryngeal and pharyngeal swelling and hematoma by a simple endoscopy. When eligible for anamneses the current sound of the voice might be helpful as well [55]. Therefore, an ORL examination should always take place because it is not possible to draw conclusions about the internal injuries from the external appearance. Strangulation attempts can be hard to evaluate. Strangulation was in 2021 the most frequent method in nearly half of all suicides (43.9%) in Germany, but in suicide attempts, strangulation is used in less than 10% [71]. The development of a step‐by‐step assessment protocol in strangulation survivors to reduce secondary injury after a nonfatal suicide attempt is encouraged [71].

Self-inflicted injuries to the head and neck region can lead to severe, life-threatening complications. ORL specialist can ensure conservative diagnostic management as well as surgical treatment of head and neck injuries. Based on our findings we recommend routine laryngoscopy in any case with possible head and neck involvement should be performed within 24h after the initial trauma. Management protocols after a suicide attempt should be established and not only validated for the psychiatry departments. In any case, a psychiatric intervention should be sought as soon as possible. Multicenter and prospective studies in the future could compensate the limitations in this study.

## Data Availability

The authors confirm that most of the data supporting the findings of this study are available within the article and further data are available on request from the corresponding author.
